# The associations between e-liquid characteristics and its pricing: Evidence from online vape shops

**DOI:** 10.1371/journal.pone.0286258

**Published:** 2023-05-26

**Authors:** Shaoying Ma, Shuning Jiang, Theodore Wagener, Darren Mays, Jian Chen, Ce Shang

**Affiliations:** 1 Center for Tobacco Research, The Ohio State University Comprehensive Cancer Center, Columbus, Ohio, United States of America; 2 Department of Computer Science and Engineering, The Ohio State University, Columbus, Ohio, United States of America; 3 Department of Internal Medicine, The Ohio State University Wexner Medical Center, Columbus, Ohio, United States of America; University of Catania, ITALY

## Abstract

Given the increase in electronic cigarette use during the past decade, the objectives of this study are to obtain comprehensive product-level information from online vape shops, which are one of the most common outlets for e-cigarette users to purchase vaping products, especially e-liquid products, and to examine the appeal of various e-liquid product attributes to consumers. We used web scraping and estimation of generalized estimating equation (GEE) models to obtain and analyze data from five popular online vape shops that sell nationwide across the US. The outcome measures are e-liquid pricing for the following e-liquid product attributes: nicotine concentration (in mg/ml), nicotine form (nicotine-free, freebase, or salt), vegetable glycerin/propylene glycol (VG/PG) ratio, and a variety of flavors. We find that the pricing for freebase nicotine and nicotine salt products are 1% (p<0.001) lower and 12% higher (p<0.001), respectively, than that for products that do not contain nicotine. For nicotine salt-based e-liquid products specifically, the pricing for a 50/50 VG/PG ratio is 10% (p<0.001) higher than the pricing for a more common 70/30 VG/PG ratio, and the pricing for fruity flavors is 2% (p<0.05) higher than that for tobacco/unflavored products. Regulating the nicotine form in all e-liquid products and fruity flavor in nicotine salt-based products will have a great impact on the market and consumers. The preference for VG/PG ratio varies by product nicotine form. More evidence on typical user patterns of a certain nicotine form (i.e., freebase or salt nicotine) is needed to assess the public health consequences of these regulations.

## Introduction

The e-cigarette market has been rapidly evolving with new configurations and product features designed to attract consumers and improve user experiences [1–5]. Several prominent features that manufacturers often manipulate include nicotine concentration, nicotine form, flavors, and the ratio of vegetable glycerin (VG) to propylene glycol (PG), or VG/PG ratio [1, 6–13]. Nicotine concentration and form are the key factors that drive the exponential growth of the e-cigarette market. Relative to freebase nicotine, products with nicotine salts use lower pH e-liquid to reduce harshness and deliver nicotine concentration at a much higher level, making them palatable yet very addictive, especially to users who are experimenting with e-cigarettes [7, 9]. Salted based nicotine e-liquids typically have 40 mg/ml or higher nicotine concentration, whereas freebase nicotine e-liquids usually have 24 mg/ml or lower nicotine level [14, 15]. In fact, the rapid takeover of the e-cigarette market by JUUL in the 2010s was largely attributable to the fact that the company revolutionized the nicotine salt form in its products.

Compared to the policy discussion on regulating nicotine in combustible tobacco, the debate is more heated for regulating nicotine levels in e-cigarettes since they have the potential to help smokers quit [16–19]. However, the concerns are that these products attract youth and prolong nicotine addiction [20]. Currently, the European Commission requires a maximum nicotine concentration of 20 mg/ml in products sold in the EU [21, 22]. Similar policies have been proposed in the US, but none of the bills have been passed to date [20, 23]. Evidence is lacking on the range and availability of nicotine concentration and form and how much these nicotine features matter in driving product popularity, which will inform the design of potential nicotine policies in the US [16, 24].

Another popular product attribute of nicotine and tobacco products is flavor, which has been shown to attract users [13, 25–28]. Various flavor restrictions have been adopted or proposed by federal, state, and local authorities to reduce product popularity or abuse liability. In April 2022, the FDA issued two proposals that would ban menthol as a characterizing flavor in cigarettes and all characterizing flavors other than tobacco in cigars. If these proposals lead to policy actions, the flavor availability in disposable e-cigarettes may incentivize the transition from combustible smoking to e-cigarette use. However, scientific evidence has also documented that flavor is a main reason that youth and young adults start to experiment with e-cigarettes, which has motivated the FDA to ban characterizing flavors other than menthol/mint and tobacco in cartridge-based e-cigarettes. The apparent exemption has since prompted growth in the sales of disposables (e.g., Puff Bar), which may have undermined the intention of the policies to reduce e-cigarette use among young people [29–33].

The importance of assessing how flavors determine product popularity is further highlighted by state and local actions. A number of states or localities, including New Jersey, New York, and San Francisco, California, have adopted local restrictions to reduce the availability of flavored e-cigarette products [34–36]. However, these flavor restrictions vary in scope or dosage (i.e., whether there are exemptions based on flavors, models, sales channels, etc.), suggesting a lack of evidence informing the best policy strategies [35, 36]. It is thus critical to assess the importance of flavors in determining product popularity, especially in the context of other e-cigarette features, which will provide insight on the impact of flavor bans or restrictions.

In terms of imposing product standards, the FDA could also regulate VG/PG ratio. The higher the VG/PG ratio, the bigger the vapor clouds and smoother the feeling on the throat when exhaled; a lower VG/PG ratio creates a sharp throat hit like cigarettes, and the vapor clouds produced are less dense [37, 38]. The availability of various VG/PG ratios in the market may result from manufacturers’ intention to attract different market segments or consumer types. Therefore, it is possible to regulate VG/PG ratios so that products in the market appeal to smokers who are using e-cigarettes to quit combustible tobacco smoking instead of young people who are experimenting with the products.

E-cigarette purchasing channels include brick-and-mortar stores (e.g., convenience stores, gas stations, grocery stores, drugstores/pharmacies, mass merchandiser outlets), specialty vape shops, and online stores [25]. Among e-cigarette users in the US, about 27% of adults, 18% of adolescents above legal age, and 25% of adolescents under legal age reported purchasing their e-cigarettes online [39, 40]. Online stores provide an easy-to-access retail environment for vapers especially during the COVID-19 pandemic [41, 42]. Existing studies also show that e-cigarette online vendors are active in marketing activities to attract customers [43, 44]. Thus, it is crucial to obtain and analyze product-level information from online stores that sell e-cigarette products.

The e-liquid data we obtained from online stores are particularly suitable for estimating the relative importance of product attributes to consumers due to the following advantages: (1) Web-scraped data do not contain self-reported data (information that is affected by measurement error) improving accurate measurement of product attributes; (2) e-cigarettes sold online are not captured by typical market data collected from brick-and-mortar stores, enabling us to capture a unique market segment; and (3) Web-scraped data capture how products are marketed to consumers using multiple flavor descriptors, more accurately measuring flavor marketing than other data sources such as packaging and labeling.

To better inform FDA policies, as well as state and local policies regulating product standards, we utilize a unique dataset of over 14,000 e-liquid products sold online to assess how e-liquid attributes (nicotine concentration, nicotine form, flavor, and VG/PG ratio) contribute to its pricing. Specifically, we test the following hypotheses: 1) nicotine salts and freebase nicotine e-liquids are both priced higher that nicotine-free e-liquids, holding other key attributes constant; 2) non-tobacco flavored e-liquids are priced higher than tobacco/unflavored e-liquids, holding nicotine forms, strength and VG/PG ratios constant; 3) certain VG/PG ratios drive higher e-liquid pricing relative to a common 70/30 VG/PG ratio, holding other e-liquid characteristics constant. We estimate the pricing for e-liquid attributes, and the higher the pricing for a certain attribute, the more important that attribute is in determining product popularity and the greater impact a regulation would have when targeting that specific attribute. This analysis can inform policies by identifying e-cigarette features that are potential regulatory targets and assessing the potential consequences of setting product standards for these features.

## Materials and methods

From February to May 2021, we obtained information for over 14,000 e-liquid products from five online stores via web scraping. Specifically, in January 2021, we searched on Google and Reddit using the key terms “best online vaping stores in 2020”. We selected three stores from top results from Google and two stores from Reddit discussions and verified that these stores sell products nationwide in the US. Five stores in our sample are all US-based, though four of them do offer international shipping to some countries. The details of the sampling strategy and data collection process were published previously [45]. In that paper, we standardized e-liquid prices using volume sizes and converted unit prices into the unit of dollar per milliliter. For the convenience of interpretation of our results here, we use US cents per milliliter as the outcome variable in the current study.

Along with coding standardized prices, we also coded product features including nicotine concentration (in mg/ml), product flavors and flavor categories (e.g., fruit/sweet, menthol, tobacco), VG/PG ratio, and whether an e-liquid product contains salt or freebase nicotine, allowing us to estimate pricing for these attributes and their relative importance. The detailed procedures of coding attributes can be found in S1 Text.

The modeling in this study takes account of within-brand correlation, and robust standard errors are clustered at brand level. There are a total of 233 brands in our sample. The brand names are publicly available through a preprint of another paper that is currently under review [46], and the brand name data will continue to be publicly accessible as an appendix when the manuscript is published.

### Measures

#### Outcome variable: Standardized prices

Standardized prices are calculated by dividing sales prices in cents (¢) by total volume in ml of a product. For each e-liquid product:

Standardizedprice=(salesprice)/(totalproductvolume) (1)

In cases where e-liquid products are sold in packs or contain multiple bottles in a unit, we use total pack volume as the denominator in Eq 1, which is the number of bottles in the e-liquid product multiplied by volume per bottle.

#### Attributes or explanatory variables

*Flavor*. Based on the distribution of flavors (presented in Table 1), we categorized flavors into the following groups: any menthol/mint flavors; sweets, not menthol/mint or fruit; nut/spice/alcohol/beverage, not menthol/mint or sweets; fruit only, no other flavors; and tobacco/unflavored (the reference group). As detailed in S1 Text, for most of the e-liquids in our sample, we were able to scrape explicit flavor information from the online stores; we also identified a list of concept flavors and linked them to explicit flavor categories [47]. Through another publication, we built a comprehensive and up-to-date semantic database of e-liquid flavors upon the e-liquid flavor wheel in the tobacco control literature, which facilitated the classification of flavor key terms in our analysis [48].

**Table 1:** Summary statistics (n = 14,407).

**Outcome variables**		
	Mean	SD^a^
Standardized prices^b^	24.5821	14.1557
Standardized prices in log form	3.0641	0.5198
**Attribute (independent) variables**		
	**Mean**	**SD**
Nicotine concentration (mg/ml)	12.2232	16.3917
	**Frequency**	**Percentage**
*Nicotine form*		
None	3,316	23.02
Freebase	6,995	48.55
Salt	4,096	28.43
*VG/PG ratio*		
70/30	7,176	49.81
50/50	1,962	13.62
75/25	591	4.10
80/20	716	4.97
Other	1,175	8.16
Missing	2,787	19.34
*Flavor*		
Tobacco/unflavored	545	3.78
Fruit, no other flavors	5,132	35.62
Sweets, not menthol or fruit	1,162	8.07
Any menthol	3,885	26.97
Nut/spice/alcohol/beverage, not menthol or sweets	3,683	25.56

^a^SD: standard deviation.

^b^Standardized price equals the ratio of sales price per ml in cents. There are 233 brands in the sample.

*Nicotine concentration and form*. We used two measures for nicotine: (1) a continuous variable of nicotine concentration in mg/ml and (2) a categorial variable to indicate whether a product had no nicotine (0 mg/ml, the reference group), used freebase nicotine, or used nicotine salt. The plot of nicotine concentration distribution can be found in S1 and S2 Figs.

*VG/PG ratio*. We categorized VG/PG ratios into the following groups: 70/30 (reference group), 50/50, 75/25, 80/20, other, and missing. We further provide the distribution plot of VG/PG ratios categorized as “other” in S3 Fig.

### Data analysis

We used generalized estimating equation (GEE) models with a log link function to estimate the pricing for different e-liquid attributes while accounting for the correlation among different products sold under the same brand (e.g., different flavors or nicotine concentrations). The regressions were carried out for the full sample (i.e., regardless of nicotine form), as well as by nicotine form stratifications (i.e., no nicotine, freebase nicotine, and salt nicotine), as nicotine form is an important factor of e-liquids that could impact user experience and/or abuse liability. The overall specification is outlined in Eq 2:

$$log\left({Price}_{ij}\right)=\beta_{0}+\beta_{1}\times {NicotineConcentration}_{ij}+\beta_{2}\times {NicotineForm}_{ij}+\beta_{3}\times {VGPG}_{ij}+\beta_{4}\times {Flavor}_{ij}+s_{j}+\varepsilon_{ij}$$ (2)

The natural log of the standardized price of an e-liquid product (in US ¢/ml) is expressed as a function of nicotine concentration, form, VG/PG ratio, and flavor(s). In the above model, i denotes product and j denotes store; s_j_ are store fixed effects; and ε_ij_ are the error terms. The coefficients of interest in Eq 2, *β*_1_ through *β*_4_, indicate the percentage change in the standardized price in response to the change in a certain product attribute, further measuring pricing for a certain attribute in the form of exp(). For instance, exp(*β*_2_) measures the ratio of pricing for a product that contains freebase or salt nicotine in relation to products containing no nicotine. The greater a coefficient is, the higher the pricing for that attribute; thus, more importance is given to that attribute.

In addition to the specifications outlined above, we also conducted sensitivity analysis by using effect coding for product attributes and by modeling brand effects using fixed and random effects. The cutoff of p value for statistical significance in our study is 0.05 (i.e., statistically significant at 5% level). We report estimated slope coefficients and robust standard errors in Table 2 (for GEE models), S1 Table (for fixed effects models) and S2 Table (for random effects models). In our analysis, observations with missing values in standardized prices, nicotine forms, nicotine levels or flavors are excluded from the analysis, and as a result, a total of 70 products are excluded. There are quite a few products (n = 2,787) with missing values in VG/PG, so we coded missing as one of the VG/PG categories, as shown in Tables 1 and 2, and S1 and S2 Tables.

**Table 2:** The effects of product attributes on prices (GEE)^a^.

Dependent variable: natural log of standardized price^b^				
	(1)	(2)	(3)	(4)
	Full sample	Nicotine free	Freebase nicotine	Salt nicotine
Nicotine concentration (mg/ml)	0.0031*** (0.0003) [1.003]		0.0030*** (0.0009) [1.003]	0.0022*** (0.0002) [1.002]
*Nicotine form*				
No nicotine	--			
Freebase	-0.0108*** (0.0020) [0.989]			
Salt	0.1211*** (0.0151) [1.129]			
*VG/PG ratio*				
70/30	--	--	--	--
50/50	0.0370*** (0.0087) [1.038]	0.0171 (0.0267) [1.017]	0.0346 (0.0335) [1.035]	0.1049*** (0.0171) [1.111]
75/25	-0.0209 (0.0190) [0.979]	-0.0016 (0.0140) [0.998]	-0.0127 (0.0134) [0.987]	-0.0248 (0.0304) [0.975]
80/20	-0.0067 (0.0174) [0.993]	0.0074 (0.0112) [1.007]	0.0030 (0.0153) [1.003]	-0.0611 (0.0460) [0.941]
Other	0.0448*** (0.0074) [1.046]	0.0223** (0.0077) [1.023]	0.0228** (0.0081) [1.023]	0.0930*** (0.0165) [1.097]
Missing	0.0124 (0.0071) [1.012]	-0.0139 (0.0078) [0.986]	-0.0182* (0.0081) [0.982]	0.0823*** (0.0171) [1.086]
*Flavor*				
Tobacco/unflavored	--	--	--	--
Fruit, no other flavors	0.0156 (0.0115) [1.016]	0.0215 (0.0177) [1.022]	0.0061 (0.0126) [1.006]	0.0241* (0.0116) [1.024]
Sweets, not menthol or fruit	0.0080 (0.0113) [1.008]	0.0205 (0.0177) [1.021]	0.0043 (0.0132) [1.004]	0.0183 (0.0132) [1.018]
Any menthol	0.0114 (0.0117) [1.011]	0.0223 (0.0177) [1.023]	0.0078 (0.0126) [1.008]	0.0227 (0.0118) [1.023]
Nut/spice/alcohol/beverage, not menthol or sweets	0.0092 (0.0119) [1.009]	0.0202 (0.0174) [1.020]	0.0017 (0.0128) [1.002]	0.0213 (0.0120) [1.022]
n	14,407	3,316	6,995	4,096

^a^We fit GEE models taking account of within-brand correlation. Regressions were estimated for the full sample, as well as by nicotine form (i.e., nicotine-free, salt-based nicotine and freebase nicotine e-liquids). Natural log of standardized price of an e-liquid product (in US cents per ml) is regressed on nicotine concentration (in mg/ml), nicotine form, VG/PG ratio, and flavor(s). Robust standard errors (adjusted for clustering at brand level) are reported in parentheses. Store fixed effects are controlled for in all specifications. Marginal effects showing changes in e-liquid prices are reported in brackets.

* *p* < 0.05,

** *p* < 0.01,

*** *p* < 0.001.

^b^Standardized price equals the ratio of sales price to product volume, times 100, i.e., (sales_price/product_volume)*100.

## Results

In Table 1, we present the summary statistics of e-liquid sales prices and product attributes. The average standardized price of e-liquids is approximately 25 ¢/ml. The average nicotine concentration is 12 mg/ml, with 23% containing no nicotine, 49% using freebase nicotine, and 28% using nicotine salt. For VG/PG ratios, 70/30 is the most prevalent (50%), followed by 50/50 (14%), 80/20 (5%), and 75/25 (4%). All other ratios together make up 8% of the sample. VG/PG ratios were unspecified (missing) in 19% of the sample. For flavor profiles, 27% of the sample list menthol/mint in their profiles; 8% list sweet flavors that do not contain additional labels of menthol/mint or fruit; 26% list nut, spice, beverage, or alcohol flavors that do not contain additional labels of menthol/mint or sweets; 36% list fruit flavors only; and 4% list tobacco flavor or do not contain any flavors in their profiles. As shown in S1 and S2 Figs, the most frequent nicotine concentrations are 6 mg/ml (24.5%), 3 mg/ml (24.44%), 0 mg/ml (23%), 50 mg/ml (7.86%), 35 mg/ml (3.85%), 48 mg/ml (2.65%), 25 mg/ml (2.58%), 12 mg/ml (2.3%), 24 mg/ml (2.19%), 30 mg/ml (1.78%), and 36 mg/ml (1.43%).

We report our main results in Table 2, with Columns 1–4 presenting analyses for the full sample, the sample of products containing no nicotine, the sample of products containing freebase nicotine, and the sample of products containing nicotine salt, respectively.

Analyses using the full sample suggest that, for e-liquid products, the pricing for freebase nicotine and nicotine salt products are 1.08% (p < 0.001) lower and 12.11% higher (p < 0.001), respectively, than that for products that do not contain nicotine. An increase in nicotine strength by 1 mg/ml raises e-liquid pricing by 0.31% (p < 0.001), when holding nicotine form, VG/PG ratio and flavor(s) constant. In addition, the pricing for a 50/50 VG/PG ratio and uncommon or “other” VG/PG ratios are 3.7% (p < 0.001) and 4.48% (p < 0.001) higher than that for a common 70/30 VG/PG ratio. We did not observe any significant associations between flavors and pricing for the full sample.

For e-liquid products that do not contain nicotine (0 mg/ml), the prices for uncommon or “other” VG/PG ratios are 2.23% (p < 0.01) higher than the prices for a common 70/30 VG/PG ratio. Flavor attribute is not significantly associated with the prices of this type of e-liquid product.

Among e-liquid products that contain freebase nicotine, an increase in nicotine strength by 1 mg/ml raises pricing by 0.3% (p < 0.001), when holding VG/PG ratio and flavor(s) constant; the prices for uncommon or “other” VG/PG ratios are 2.28% (p < 0.01) higher than the prices for a common 70/30 VG/PG ratio. Flavor attribute is not significantly associated with the prices of this type of e-liquid product.

For nicotine salt-based e-liquid products, the prices for a 50/50 VG/PG ratio and for uncommon or “other” VG/PG ratios are 10.49% (p < 0.001) and 9.3% (p < 0.001) higher, respectively, than the price for a common 70/30 VG/PG ratio. In addition, the prices for fruity flavors are 2.41% (p < 0.05) higher than that for tobacco/unflavored products. Among this type of e-liquids, an increase in nicotine strength by 1 mg/ml raises pricing by 0.22% (p < 0.001), when holding VG/PG ratio and flavor(s) constant.

To test the sensitivity and validate our results, we estimated fixed-effect and random-effect models, as shown in S1 and S2 Tables, respectively. The results are mostly consistent with what is shown in Table 2, and the conclusions remain largely similar. We also conducted analyses using the full sample with effect coding of attributes, and the corresponding relative importance estimation is reported in S4 Fig. In terms of relative importance, the most important attribute is nicotine form (64.28%), followed by VG/PG ratio (32.02%) and flavor (3.7%).

## Discussion

This research is the first to utilize web data of e-liquid products collected from online vape shops to assess the importance of e-liquid product attributes to inform regulatory priorities and potential product standards. Our study also generates novel data on e-liquid products because existing sources of e-cigarette product attributes primarily come from brick-and-mortar stores (i.e., Nielsen Retail Scanner data), which may not capture the full spectrum of the e-cigarette market, particularly e-liquid products, which are often sold in vape shops or online.

Using a hedonic pricing model, we estimate the pricing of various e-liquid attributes, including nicotine concentration, form, flavor, and VG/PG ratio. These attributes are either regulated or being considered for regulation by federal, state, and local authorities in the US. Given that prices are determined by the supply and demand sides of an imperfectly competitive market, such as the e-cigarette market. On the demand side, market prices reflect consumers’ preferences for these attributes and what would happen if these attributes were restricted or removed by regulators. On the supply side, market prices may reflect the production costs of products that have a certain feature or attribute, or that the product can be sold at a higher price with a higher profit margin.

Our findings suggest that nicotine form is the key and most important factor of e-liquid pricing and drives prices more significantly than other attributes such as flavors and VG/PG ratios. Although products containing no nicotine or freebase nicotine constitute the majority of e-liquid products (72%), products that contain nicotine salt (28%) are priced higher. This finding can be interpreted based on both supply and demand side considerations: It is likely that nicotine salt products bear a higher production cost, or a higher profit margin, compared to other products and that consumers prefer salt products to other products and pay a higher price to obtain them.

If our findings reflect consumer preferences, the results suggest that regulating e-cigarette nicotine form and strength may have a greater impact on e-cigarette users who use e-liquid, compared to regulations on other features. This conclusion complements a recent study published by Ali et al., (2023) which compared e-cigarette sales in states that restrict nicotine strength with states with no restrictions and found that nicotine strength restrictions are associated with reductions in mean nicotine strength and the sales of products sold in brick-and-mortar stores (e.g., disposables, pod systems, etc.,) [47]. In summary, nicotine form, and therefore strength, is an important factor that drives consumer preference or sales across retail channels (online vape shops and brick-and-mortar stores) and product types, indicating that regulations on these features (e.g., setting nicotine concentration or strength caps) could have a great impact on behaviors and public health.

Nevertheless, regulations on nicotine form and strength may need to balance both the harm-reducing and harm-inducing uses of e-cigarettes by weighing potentials to reduce youth addiction vs. harm-reducing transitions to e-cigarettes. Given that youth and young e-cigarette users prefer either pod systems (e.g., JUUL) or disposables (e.g., puff bars) to e-liquid products, our findings on nicotine salt preference may be particularly relevant to adult e-liquid or open-system e-cigarette users [49, 50]. In addition, existing evidence shows that open systems may help adult smokers to quit smoking [51]. The combined evidence therefore suggest that, although the marketed growth of JUUL and puff bar use among youth and young adults clearly demonstrates the potential of adopting nicotine form or strength restrictions to reduce youth addiction, [49, 51] such policies could also discourage smokers who prefer to use nicotine salt products for quitting. A comprehensive cost-benefit assessment is therefore needed to guide nicotine-related policies.

In our study, we did not find flavors to be significantly associated with e-liquid prices. This is partly due to our focus on e-liquid products sold online, where products with flavors other than tobacco/unflavored account for 96% of the market. Therefore, our sample may not have sufficient statistical power to detect what would happen if flavors other than tobacco and menthol were removed from e-liquid products. Nevertheless, the high prevalence of fruit and other flavors in e-liquid products further highlights the loopholes of the 2020 FDA ban on flavored cartridge-based e-cigarettes (other than tobacco- or menthol-flavored ones), which allows flavored disposables and open-system, refillable e-liquid products to remain in the market [52–54].

Nonetheless, when we focus on nicotine salt-form e-liquid products, we found fruity flavors to be significantly associated with prices. Given that e-liquid products are mostly preferred by adults and not by youth and young adults, the heterogeneity in flavor preferences by nicotine form may reflect differences in how adult e-liquid users intend to use the products. Some evidence suggests that adults who use e-cigarettes to quit smoking might prefer fruity flavors [55]. However, it is important to note that adults who do not smoke or are dual users of e-cigarettes and e-cigarettes and are not trying to quit smoking also use flavored e-cigarettes [56]. Thus, fruity flavors might be used for harm reduction (helping to quit smoking) among a subset of adult e-cigarette users; among other adults, flavored e-cigarettes might promote e-cigarette use and induce related harms. It is again crucial to balance the benefits and risks of flavored e-cigarettes: fruity flavors might be helpful for some adults or youth trying to quit smoking, but they could also be harmful as they contribute to the initiation and long-term use of e-cigarettes [57–59]. The fact that few unflavored e-liquids available in our sample could also reflect that flavors are key drivers of consumer preferences, and thus manufacturers offer few units of tobacco-flavored or unflavored e-liquids.

We also estimated the associations between VG/PG ratios and e-liquid pricing by nicotine form. For e-liquid products that do not contain nicotine or are freebase, uncommon VG/PG ratios are associated with higher prices, suggesting e-liquids with these ratios may appeal to a specific subgroup of consumers. For salt nicotine products, both a 50/50 ratio and uncommon VG/PG ratios are associated with higher prices, relative to a 70/30 ratio; this might indicate that salt-form e-liquid users are not as interested in vaping bold or thick clouds as users of nicotine-free or freebase nicotine products. It is also likely that products with uncommon VG/PG ratios have higher production costs due to the lack of economies of scale (i.e., lower costs per unit are associated with increasing production).

In our modeling, prices are taken as the outcomes. Therefore, we are not able to assess the importance of prices in relation to non-price attributes such as nicotine and flavor. Nonetheless, we found the form of nicotine-salt is significantly associated with higher prices relative to nicotine-free, which may inform the design of e-cigarette tax bases. Thus far, several bills proposing federal e-cigarette taxes suggest using nicotine content levels as tax bases. Our findings indicate that, if we indeed tax e-cigarettes based on nicotine concentration, adult consumers of e-liquid products may be discouraged from using salt products, which could hinder their transitions from cigarettes to e-cigarettes. Balancing the harm-inducing and harm-reducing use of e-cigarettes will be the key determinant of how to best design e-cigarette taxes. Furthermore, given that salt products are already priced higher, ad valorem taxes based on wholesale or retail prices (e.g., 50% of wholesale prices) will impose higher taxes per unit on salt products compared to specific or volume-based taxes. Nicotine content-based taxes therefore may not be necessary in places with an ad valorem tax base to achieve a high tax burden on salt products.

Finally, in January 2021, the FDA issued warnings to companies that produced and sold e-liquid products online without a premarket tobacco product application (PMTA) by the September 9, 2020 deadline. However, when we obtained online store data from February to May 2021, 240 different e-liquid brands remained on the market, and only 23% of over 14,000 unique products did not contain nicotine. Therefore, some of the products that we assessed in this study may be illegal in the US market. Future follow ups on brand availability in online stores will provide data on the enforcement of PMTA requirements and how the process impacts the e-cigarette market and product attributes.

There are several limitations of this study that we hope to address in the future. First, the focus of the study is on e-liquid products, a segment of the e-cigarette market. In the future, we will examine a more comprehensive list of products that additionally includes cartridge-based and disposable products. Second, we assume that the e-liquid market is close to a competitive market and the equilibrium prices reflect both the demand-side consumers’ preference and the supply-side production costs and profit margins. However, as long as e-liquid products are produced and marketed at higher prices, their valuation is higher from a marketing perspective. In other words, a segment of consumers is willing to pay higher premiums for the identified product features, especially for salt products that account for 28% of the total products marketed in our online store data. Third, although we have developed algorithms to identify synthetic or natural nicotine, the prevalence of synthetic nicotine among e-liquid products was very low (2.13%). Therefore, we did not include this feature in the regression. Another key factor of e-liquid pricing is the cost of resources invested to assess and ensure the safety of e-liquid products. Despite that many manufacturers of e-liquid products invest little to nothing in research to ensure the safety of their products, there is a growing awareness towards the importance of product safety [60]. Investments to ensure the quality of raw materials and verify the safety of final products can significantly affect the final prices of e-liquid products, and these costs are not accounted for in our analysis of e-liquid pricing. Finally, although we have over 14,000 unique products in our data, they come from a convenience sample of five online stores. In the future, we will use organic traffic to a website to better design sampling strategies for online store data collection.

## Conclusions

Our study presents evidence of relative appeal of nicotine forms as well as fruity flavors of salt-based e-liquids to e-cigarettes users, using a convenience sample of five online stores, with over 14,000 unique products in our data. The reported results have policy implications for potential FDA regulatory actions such as ban on certain flavors, and restrictions on nicotine levels and forms. We focus on e-liquid products only and assume a competitive e-liquid market so that the equilibrium prices reflect consumers’ preferences. The reported results also provide an assessment of the perception of relative importance of various product attributes among e-cigarette users, and the likely impacts of those attributes on the future use of e-cigarettes.

## Supporting information

S1 Fig Distribution of nicotine concentration in mg/ml (n = 14,407). For each sector of the pie chart, the label contains two numbers, the first is nicotine level (mg/ml) and the second is percentage of products with that specific nicotine level; “other” category includes the following nicotine levels: 2 mg/ml, 4 mg/ml, 5 mg/ml, 9 mg/ml, 15 mg/ml, 18 mg/ml, 20 mg/ml, 28 mg/ml, 40 mg/ml, 45 mg/ml, 55 mg/ml, 59 mg/ml, 60 mg/ml; see S2 Fig for distribution of nicotine concentration in the “other” category.
(TIF)

S2 Fig Distribution of nicotine concentration in the “other” category (unit: mg/ml; n = 14,407). This figure shows the distribution of nicotine levels that are relatively rare among the e-liquids in our sample; the horizontal axis shows nicotine level (mg/ml), and the vertical axis shows percentage of products with that specific nicotine level; for the commonly used nicotine levels in our sample, see S1 Fig.
(TIF)

S3 Fig Distribution of VG/PG in the “other” category (n = 14,407). The figure shows the distribution of VG/PG ratios that are relatively rare among the e-liquids in our sample; the horizontal axis shows VG/PG ratio, and the vertical axis shows percentage of products with that specific VG/PG ratio; “other” category includes the following VG/PG ratios: 60/40, 100/0, 65/35, 30/70, 90/10, 85/15, 20/80, 38/62, 40/60, 70/20, 40/50; product VG/PG information were extracted from store websites and it is possible that online stores mislabeled few products as “70/20” (0.02%) and “40/50” (0.01%); for the commonly used VG/PG ratios, see Table 1.
(TIF)

S4 Fig Relative importance measured using e-liquid pricing. This figure shows the estimated relative importance of three product attributes (nicotine form, VG/PG ratio, and flavor) in determining e-liquid pricing.
(TIF)

S1 Table The effects of product attributes on prices (brand fixed effects). (PDF)

S2 Table The effects of product attributes on prices (brand random effects). (PDF)

S1 Text The coding of product attributes. (PDF)

S1 Dataset Online vape shop data used in the analysis. (XLS)
