# Supplementary figures and images for: The associations between e-liquid characteristics and its pricing: Evidence from online vape shops
Source: PLoS One. 2023 May 26;18(5):e0286258. doi: 10.1371/journal.pone.0286258 (PMC10218732; doi:10.1371/journal.pone.0286258)

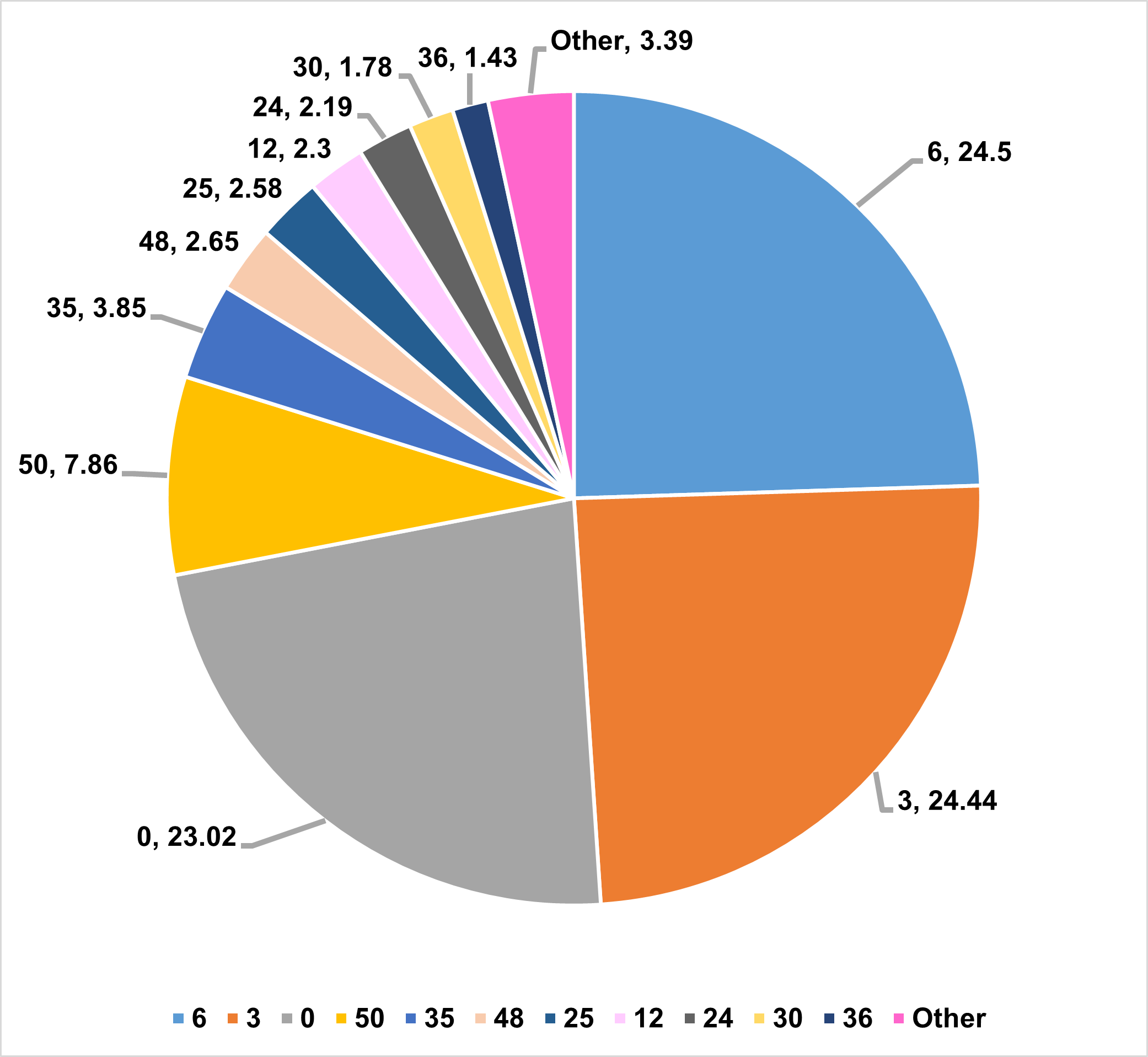

Supplement: S1 Fig — For each sector of the pie chart, the label contains two numbers, the first is nicotine level (mg/ml) and the second is percentage of products with that specific nicotine level; “other” category includes the following nicotine levels: 2 mg/ml, 4 mg/ml, 5 mg/ml, 9 mg/ml, 15 mg/ml, 18 mg/ml, 20 mg/ml, 28 mg/ml, 40 mg/ml, 45 mg/ml, 55 mg/ml, 59 mg/ml, 60 mg/ml; see S2 Fig for distribution of nicotine concentration in the “other” category. (TIF) [file pone.0286258.s001.tif]

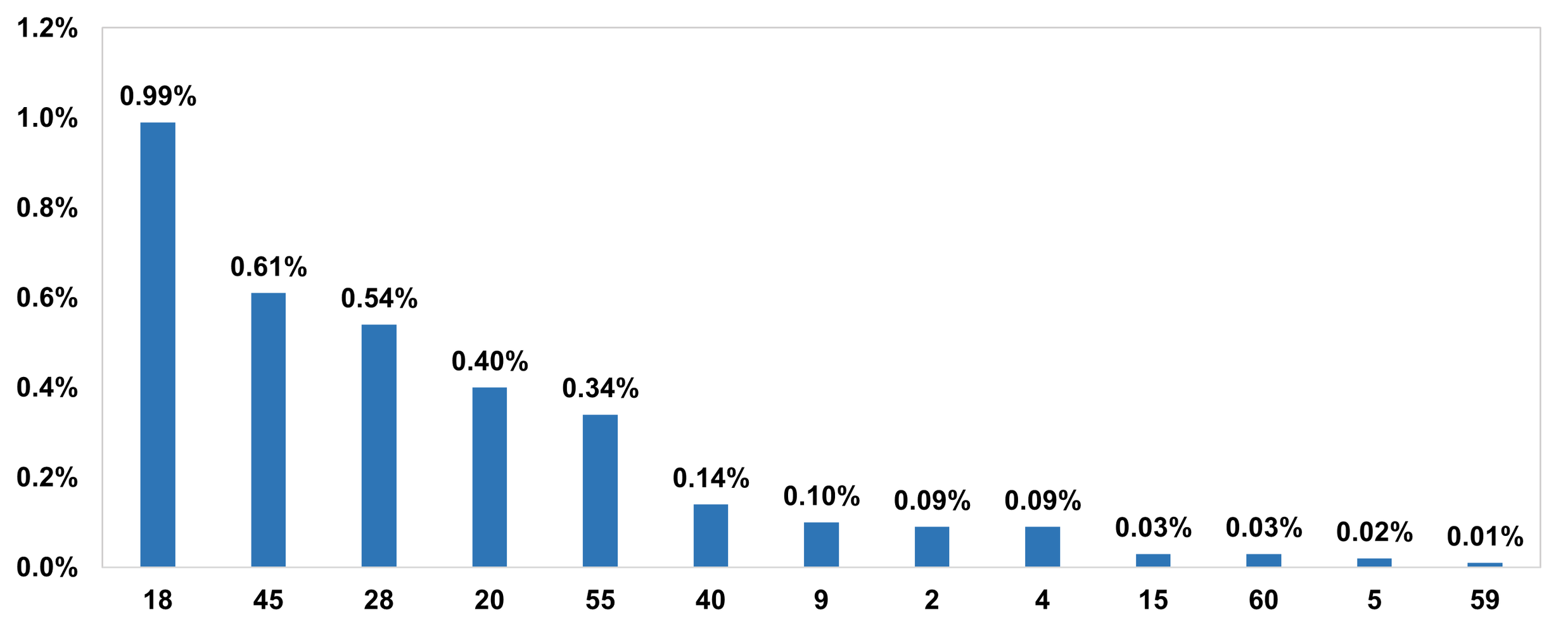

Supplement: S2 Fig — This figure shows the distribution of nicotine levels that are relatively rare among the e-liquids in our sample; the horizontal axis shows nicotine level (mg/ml), and the vertical axis shows percentage of products with that specific nicotine level; for the commonly used nicotine levels in our sample, see S1 Fig. (TIF) [file pone.0286258.s002.tif]

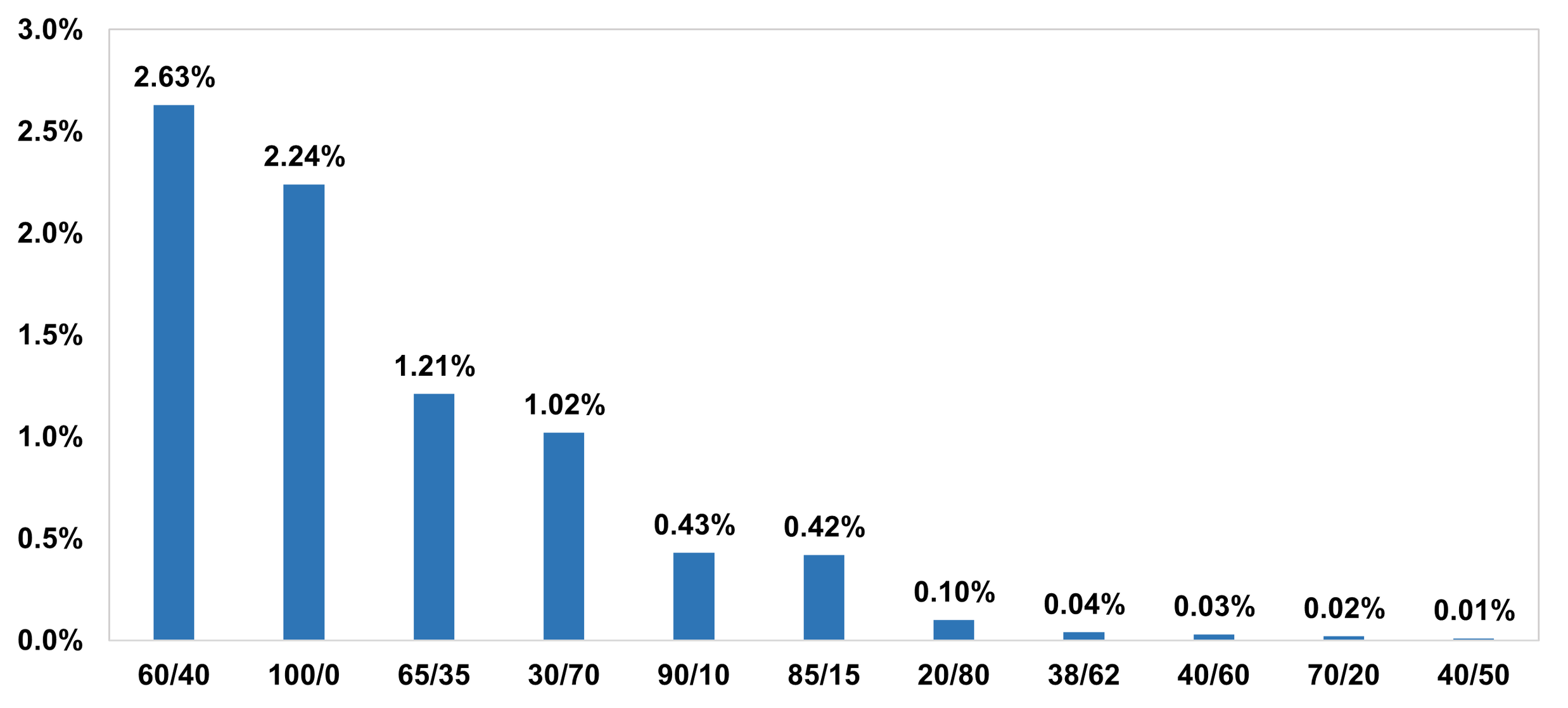

Supplement: S3 Fig — The figure shows the distribution of VG/PG ratios that are relatively rare among the e-liquids in our sample; the horizontal axis shows VG/PG ratio, and the vertical axis shows percentage of products with that specific VG/PG ratio; “other” category includes the following VG/PG ratios: 60/40, 100/0, 65/35, 30/70, 90/10, 85/15, 20/80, 38/62, 40/60, 70/20, 40/50; product VG/PG information were extracted from store websites and it is possible that online stores mislabeled few products as “70/20” (0.02%) and “40/50” (0.01%); for the commonly used VG/PG ratios, see Table 1. (TIF) [file pone.0286258.s003.tif]

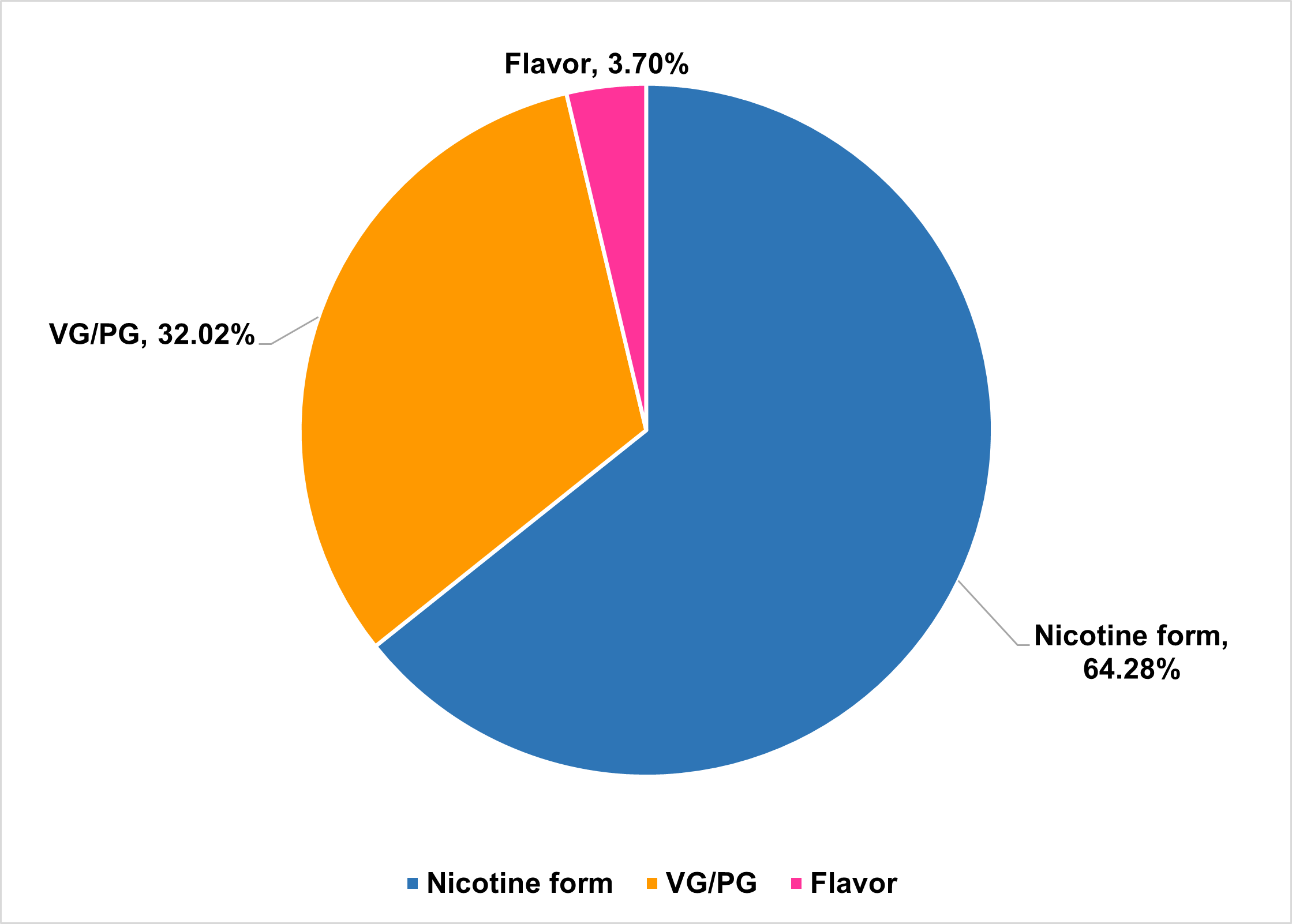

Supplement: S4 Fig — This figure shows the estimated relative importance of three product attributes (nicotine form, VG/PG ratio, and flavor) in determining e-liquid pricing. (TIF) [file pone.0286258.s004.tif]
