# Supplementary material for: The associations between e-liquid characteristics and its pricing: Evidence from online vape shops
Source: PLoS One. 2023 May 26;18(5):e0286258. doi: 10.1371/journal.pone.0286258 (PMC10218732; doi:10.1371/journal.pone.0286258)
Supplement: S1 Table — (PDF) [file pone.0286258.s005.pdf]

S5 Table. The effects of product attributes on prices (brand fixed effects)[a]

Dependent variable: natural log of standardized price[b]	(1) Full sample	(2) Nicotine free	(3) Freebase nicotine	(4) Salt nicotine
Nicotine concentration (mg/ml)	0.0115***		0.0075**	0.0078***
	(0.0010)		(0.0028)	(0.0008)
Nicotine form				
None	--			
Freebase	-0.0455***			
	(0.0061)			
Salt	0.3359***			
	(0.0485)			
VG/PG ratio				
70/30	--	--	--	--
50/50	0.1339***	0.0102	0.0835	0.3680***
	(0.0287)	(0.0703)	(0.1066)	(0.0555)
75/25	-0.0591	-0.0279	-0.0531	-0.0640
	(0.0540)	(0.0396)	(0.0398)	(0.0939)
80/20	-0.0164	0.0207	0.0092	-0.1413
	(0.0492)	(0.0331)	(0.0442)	(0.1322)
Other	0.1306***	0.0625**	0.0654**	0.3276***
	(0.0230)	(0.0223)	(0.0241)	(0.0553)
Missing	0.0263	-0.0326	-0.0530*	0.2849***
	(0.0206)	(0.0212)	(0.0224)	(0.0566)
Flavor				
Tobacco/unflavored	--	--	--	--
Fruit, no other flavors	0.0390	0.0567	0.0117	0.0884*
	(0.0372)	(0.0528)	(0.0372)	(0.0445)
Sweets, not menthol or fruit	0.0191	0.0524	0.0073	0.0902
	(0.0365)	(0.0523)	(0.0386)	(0.0486)
Any menthol	0.0286	0.0544	0.0150	0.0818
	(0.0380)	(0.0529)	(0.0375)	(0.0454)
Nut/spice/alcohol/beverage, not menthol or sweets	0.0206	0.0512	-0.0021	0.0788
	(0.0384)	(0.0520)	(0.0378)	(0.0456)
n	14,407	3,316	6,995	4,096

[a]We fit fixed effects (FE) models, and regressions were estimated for the full sample, as well as by nicotine form (i.e., nicotine-free, salt-based nicotine and freebase nicotine e-liquids). Natural log of standardized price of an e-liquid product (in US ¢/ml) is regressed on nicotine concentration (in mg/ml), nicotine form, VG/PG ratio, and flavor(s). Robust standard errors (adjusted for clustering at brand level) are reported in parentheses. Store fixed effects are controlled for in all specifications. * $p < 0.05$, ** $p < 0.01$, *** $p < 0.001$. [b]Standardized price equals the ratio of sales price to product volume, times 100, i.e., (sales_price/product_volume) × 100.
