# Supplementary material for: The associations between e-liquid characteristics and its pricing: Evidence from online vape shops
Source: PLoS One. 2023 May 26;18(5):e0286258. doi: 10.1371/journal.pone.0286258 (PMC10218732; doi:10.1371/journal.pone.0286258)
Supplement: S2 Table — (PDF) [file pone.0286258.s006.pdf]

S6 Table. The effects of product attributes on prices (brand random effects)[a]

Dependent variable: natural log of standardized price[b]	(1) Full sample	(2) Nicotine free	(3) Freebase nicotine	(4) Salt nicotine
Nicotine concentration (mg/ml)	0.0115***		0.0082**	0.0079***
	(0.0010)		(0.0027)	(0.0008)
Nicotine form				
No nicotine	--			
Freebase	-0.0455***			
	(0.0061)			
Salt	0.3346***			
	(0.0484)			
VG/PG ratio				
70/30	--	--	--	--
50/50	0.1328***	0.0265	0.0854	0.3646***
	(0.0286)	(0.0716)	(0.1038)	(0.0543)
75/25	-0.0567	-0.0176	-0.0473	-0.0622
	(0.0541)	(0.0388)	(0.0390)	(0.0928)
80/20	-0.0158	0.0208	0.0097	-0.1553
	(0.0489)	(0.0324)	(0.0437)	(0.1319)
Other	0.1301***	0.0626**	0.0656**	0.3214***
	(0.0229)	(0.0221)	(0.0240)	(0.0538)
Missing	0.0260	-0.0353	-0.0522*	0.2796***
	(0.0205)	(0.0210)	(0.0223)	(0.0552)
Flavor				
Tobacco/unflavored	--	--	--	--
Fruit, no other flavors	0.0410	0.0594	0.0137	0.0884*
	(0.0365)	(0.0503)	(0.0366)	(0.0434)
Sweets, not menthol or fruit	0.0212	0.0562	0.0095	0.0799
	(0.0358)	(0.0500)	(0.0382)	(0.0473)
Any menthol	0.0311	0.0592	0.0175	0.0828
	(0.0372)	(0.0503)	(0.0369)	(0.0443)
Nut/spice/alcohol/beverage, not menthol or sweets	0.0230	0.0545	0.0003	0.0786
	(0.0377)	(0.0494)	(0.0372)	(0.0446)
n	14,407	3,316	6,995	4,096

[a]We fit generalized least squares (GLS) random effects (RE) models, and regressions were estimated for the full sample, as well as by nicotine form (i.e., nicotine-free, salt-based nicotine and freebase nicotine e-liquids). Natural log of standardized price of an e-liquid product (in US cents per ml) is regressed on nicotine concentration (in mg/ml), nicotine form, VG/PG ratio, and flavor(s). Robust standard errors (adjusted for clustering at brand level) are reported in parentheses. Store fixed effects are controlled for in all specifications. * $p < 0.05$, ** $p < 0.01$, *** $p < 0.001$. [b]Standardized price equals the ratio of sales price to product volume, times 100, i.e., (sales_price/product_volume)*100.
