# Supplementary material for: The associations between e-liquid characteristics and its pricing: Evidence from online vape shops
Source: PLoS One. 2023 May 26;18(5):e0286258. doi: 10.1371/journal.pone.0286258 (PMC10218732; doi:10.1371/journal.pone.0286258)
Supplement: S1 Text — (PDF) [file pone.0286258.s007.pdf]

S7 Text. The coding of product attributes

Specifically, we obtained information of these non-price attributes by web scraping text from (1) product description boxes; (2) website filters (e.g., select a flavor or nicotine concentration); and (3) fixed locations to describe product information such as brand names. Within this data, we conducted keyword matching using algorithms to automatically code flavor, salt form, VG/PG ratio, and nicotine concentration. The figure on the right shows an example of online product description text that contained information about e-liquid VG/PG ratio and flavors.

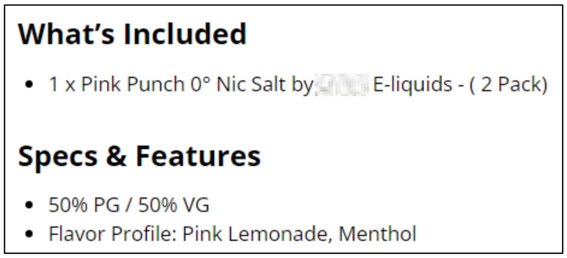

Among these attributes, nicotine concentration (in mg or mg/ml) and VG/PG ratio (e.g., 70/30) were relatively easy to identify since they contain numbers and often are clearly labeled in the text. In contrast, coding flavors and identifying whether the nicotine form of a product was salt required several steps. Specifically, in order to determine whether the nicotine form of an e-liquid product was salt, we used key-term matching to identify products with the word "salt" in their product name or product description page. We then checked the classifications to make sure that the word "salt" was associated with nicotine and not with other terms such as "sea salt."

Similarly, we employed several data curation processes to identify flavors and classify the wide spectrum of flavors into manageable and meaningful categories. First, we consolidated two sources to identify and classify flavors: (1) WordNet, a lexical database of English words, and (2) flavor wheel, a flavor word database published in the journal *Nicotine & Tobacco Research* in 2019.[1,2] We further utilized descriptive text and identified words with implicit flavor cues (i.e., concept flavors), which were added to the semantic database. By consolidating different databases and enriching the dictionary with concept flavor terms, we were able to generate a flavor semantic database that is, to our knowledge, the most comprehensive one available.[3] Next, we performed keyword matching using this comprehensive flavor semantic database, and identified flavors belonging to the following aggregated-level flavor categories: sweet/dessert, nut,

spice, alcoholic beverages, non-alcoholic beverages, menthol/mint, tobacco, and others. When we scraped e-liquid flavor information from the online stores, in most cases explicit flavor(s) of the products were provided by the online vape shops, such as strawberry and menthol. We also identified the following concept flavors:

Concept fruit: tropical, tropic

Concept menthol: green, icy, ice, iced, ice-cold, fresh, refreshing, cool, cooled, crystal, chill

Concept tobacco: natural, classic, pure

Any of the above key terms was thus linked to an explicit flavor category, and in our analysis, e-liquid flavors were classified based on the explicit flavor categories that they belonged to. In other words, an e-liquid described as “ice” (i.e., concept menthol) will be classified as menthol category.

References

1 Princeton University. About WordNet. 2010.https://wordnet.princeton.edu/
2 Krüsemann EJZ, Boesveldt S, De Graaf K, *et al.* An E-Liquid Flavor Wheel: A Shared Vocabulary Based on Systematically Reviewing E-Liquid Flavor Classifications in Literature. *Nicotine Tob Res* 2019;**21**:1310–9. doi:10.1093/NTR/NTY101
3 Ma S. Expanding the E-Liquid Flavor Wheel: Classification of Emerging E-Liquid Flavors in Online Vape Shops. *Int J Environ Res Public Health* 2022;**19**:13953. doi:10.3390/ijerph192113953
